# Modern technologies for retinal scanning and imaging: an introduction for the biomedical engineer

**DOI:** 10.1186/1475-925X-13-52

**Published:** 2014-04-29

**Authors:** Boris I Gramatikov

**Affiliations:** 1 Laboratory of Ophthalmic Optics, Wilmer Eye Institute, Johns Hopkins University School of Medicine, 600 N. Wolfe St., Baltimore MD 21287, USA

**Keywords:** Retinal scanning, Scanning laser ophthalmoscopy, Optical coherence tomography, OCT, Scanning laser polarimetry

## Abstract

This review article is meant to help biomedical engineers and nonphysical scientists better understand the principles of, and the main trends in modern scanning and imaging modalities used in ophthalmology. It is intended to ease the communication between physicists, medical doctors and engineers, and hopefully encourage “classical” biomedical engineers to generate new ideas and to initiate projects in an area which has traditionally been dominated by optical physics. Most of the methods involved are applicable to other areas of biomedical optics and optoelectronics, such as microscopic imaging, spectroscopy, spectral imaging, opto-acoustic tomography, fluorescence imaging etc., all of which are with potential biomedical application. Although all described methods are novel and important, the emphasis of this review has been placed on three technologies introduced in the 1990’s and still undergoing vigorous development: Confocal Scanning Laser Ophthalmoscopy, Optical Coherence Tomography, and polarization-sensitive retinal scanning.

## Introduction

In the past few decades, the use of light has played an important role in revealing structural and functional information from the human retina in a non-destructive and non-invasive manner. Ophthalmic optics as an active research area has been expanding steadily, providing scientists and doctors with priceless multidisciplinary information and enabling new diagnostic and therapeutic methods. New scanning and imaging technologies have had a tremendous impact on ophthalmology, where information about the fovea and the optic nerve is essential.

### The anatomy of the human eye and its optical properties

**Figure 1:**
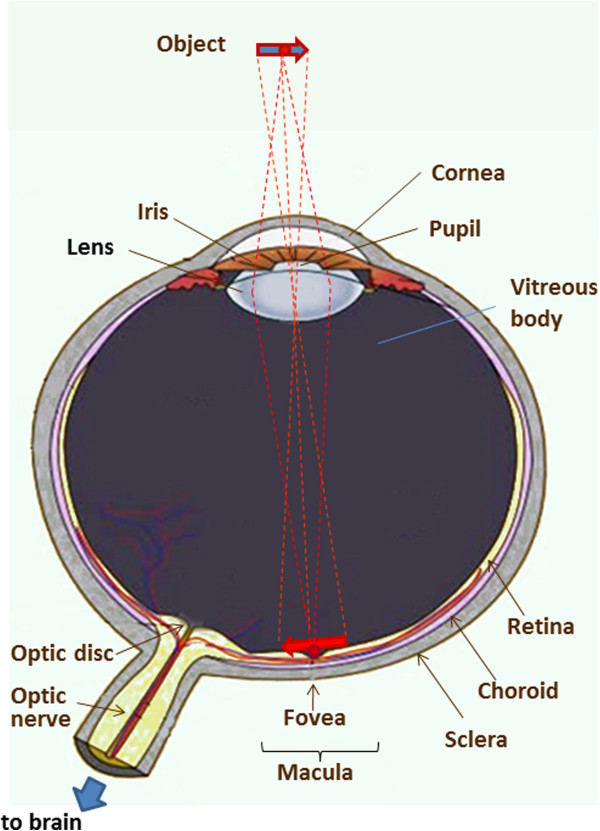
**The human eye.** The object being observed is projected onto the macula, the central part of which is the fovea, the spot of the sharpest vision.

The anatomy of the human eye is shown in Figure 1. The eyeball measures about 24 mm in diameter and is filled with jelly-like vitreous humor. The light entering the eye passes through the iris and the pupil, is focused by the cornea and the crystalline lens onto the retina in the region of the macula, its most sensitive part being the fovea, which is the spot of the sharpest vision. The retina converts the photon energy of the incoming light into electrical activity, which is transferred to the optic disc and along the optic nerve to the brain. The fibers carrying the electric signal from the fovea to the optic disc are called Henle fibers in the vicinity of the fovea, and form the thicker retinal nerve fiber layer (RNFL), the axons of the nerve fibers, mainly in the area surrounding the optic nerve. Both the Henle fibers and the RNFL change the polarization state of light – an optical property known as *birefringence*[1-5]. Birefringent materials delay the vertical (s-) and the horizontal (p-) components of light differently, and hence have a refractive index that depends on the polarization state and propagation direction of the impinging light. These optically anisotropic materials exhibit different indices of refraction for p- and s-polarization of the incoming light. Upon reflection by diffuse birefringent reflectors, such as the fovea and the optic disc, the p- and the s-components are delayed differently, yet they can be detected separately in a polarization-sensitive (PS) detection system. The thickness of the RNFL is not constant over the retina. Another part of the human eye that is birefringent is the cornea, with its corneal collagen fibrils in fact constituting the main part of the birefringence of the eye, ca. six times higher than the birefringence of the fovea. It has also been shown that corneal birefringence varies greatly among people and, within a single cornea, significantly with position [6]. The layer underneath the retina is called the choroid, which is just above the sclera. The choroid contains numerous tiny blood vessels responsible for the retina’s metabolism. Deeper layers of the retina can today be examined with new technologies, most of which are based on scanning the fundus of the eye. They can be polarization-insensitive, or polarization-sensitive. Both types will be discussed in the upcoming sections.

### Fundus photography

Fundus photography was introduced in the 1920’s and has been used extensively since the 1960’s – first as a standard photographic technique based on 35 mm film, and later as digital photography. Of main interest is the optic nerve photography, allowing the evaluation of structural relationships within the nerve. It also allows the practitioner to examine fine details not easily seen on examination, as well as evolution of such changes over time. Additional techniques such as stereo disc photography and red-free RNFL photography led to substantial enhancement of fundus photography.

In analogy with the indirect ophthalmoscope, the objective lens forms a real intermediate image of the illuminated fundus in front of a pinhole mirror. Behind the pinhole mirror, a second intermediate image is formed by the main objective lens. With a movable focusing lens, the rays are parallelized, thus enabling the use of high-resolution cameras. The maximum resolution of fundus cameras is considered to be ca. 6 μm, but it can only be obtained for a small field of view (FOV), and if the pupil is dilated. To capture reflection-free fundus images, with a large FOV, a small aperture stop is needed, which, in turn, reduces the resolution (to approximately 10 μm for a FOV of 50°. Normally, the maximum FOV for a fundus camera is 50°. Only with special mydriatic (for work with pupil dilation) cameras, a larger FOV of up to 60° can be realized. Typical FOV graduations are 20° to 50°. Peripheral areas of the retina which lie outside the central FOV can be registered when the patient looks in different directions, changing the line of sight. With special *Auto Mosaic* (or *Montage*) software, the individual images can then be stitched together forming a panoramic image which can span an angular range of up to 110°. Table 1 shows a comparison between fundus photography and other retinal imaging technologies, with respect to FOV, resolution, and size of the features of interest. It can be seen that the large FOV with fundus cameras comes at a cost of lower resolution and inability to detect microscopic structures, such as very small blood vessels, cone photoreceptors etc. Also, no information from deeper retinal layers can be obtained. A good comparative analysis of fundus camera systems has been reported in [7].

**Table 1:** Comparison between retinal imaging technologies

**Technology**	**Field of View (FOV) in angular degrees**	**Resolution in μm**	**Detectable features of interest**
Fundus photography	20°…30°…50° (60°) (up to 110° with *Montage* Software)	ca. 10 μm (lateral); Depends on the FOV	Optic disc, macula, posterior pole, retinal blood vessels, drusen, pigmentation, fluorescein angiography
Hyperspectral Imaging (HIS)	7…20°	Similar to fundus photography	Retinal blood vessels, (oxygen saturation), macular pigment, optic disc drusen
Confocal Scanning Laser Ophthalmoscope (cSLO)	5…25°	5-10 μm lateral 20–50 μm axial (distance between slices)	Drusen, microvascular angiopathy, nerve fiber bundles, angioscotomas
Adaptive Optics Scanning Laser Ophthalmoscope (AOSLO)	1°…8°	1.5…3 μm lateral less than cone-to-cone spacing; depends on motion stabilization	Individual cone photoreceptors (diameter 5–7 μm)
Scanning Laser Polarimeter (SLP)	40° x 20°	46 μm lateral	Retinal nerve fiber layer thickness around the optic disc
Optical Coherence Tomography (OCT)	5°…15°	3…10 μm lateral (depends on the the numerical aperture) 2…10 μm axial (depends on the bandwidth of the source and the axial scan speed)	Microscopic structures in intra-retinal layers, choroidal vessel system,
Polarisation Sensitive Optical Coherence Tomography (PS OCT)	20°…40°	5…20 μm lateral (depends on the the numerical aperture) 10–12 μm axial (depends on the bandwidth of the source and the axial scan speed)	Tissue organization at the molecular level, retinal pigment epitelium (polarization scrambling), drusen, Bruch’s membrane, retinal ganglion cells
Retinal Birefringence Scanning (RBS)	3°… 20°	Depends on the sampling rate	Fovea, optic nerve

**Figure 2:**
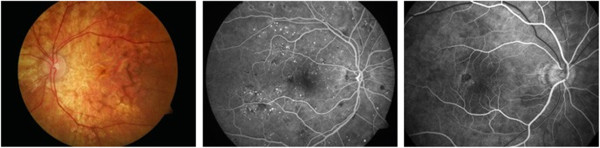
**Fundus images taken with the FF450 Fundus Camera from Carl Zeiss Meditec, Inc.** Left: color image; Middle: fluorescein angiography image; Right: zoom in the macular region. Courtesy of Carl Zeiss Meditec, Inc.

The cost of fundus photography continues to be significantly lower than the newer techniques based on retinal scanning. Its main advantages are the easy interpretation, full color (helping to distinguish between cupping and pallor), better detection of disc hemorages, peripapillary atrophy etc. Disadvantages include lack of quantitative description and hence inter-observer variability, need of highest photographic quality (not always easily achievable), and difficult serial comparison because of limited ability to detect subtle changes with a photograph. Another drawback of fundus photography is the need of high light intensity for illumination of the retina, in the order of 10-100% of the maximum permissible levels [8], typically delivered by a flash. Figure 2 shows three fundus images taken with the FF450 Fundus Camera from Zeiss, whose standard configuration is equipped for color imaging, fluorescein angiography and filter-based red-free, red and blue imaging, courtesy of Carl Zeiss Meditec.

### Hyperspectral imaging of the fundus

Hyperspectral imaging (HSI) originated from remote sensing and has been explored for various applications by NASA. It is an emerging imaging modality for medical applications [9]. HSI acquires a three-dimensional dataset called hypercube, with two spatial dimensions and one spectral dimension. In biology and medicine it is being used in image-guided surgery, tissue optics, cancer diagnostics, kidney disease, retinal diagnostics etc. HSI can deliver nearly real-time images of biomarker information, such as oxyhemoglobin and deoxyhemoglobin, providing assessment of tissue pathophysiology based on the spectral characteristics of different tissue. HSI has been successfully applied to the diagnosis of hemorrhagic shock, the assessment of peripheral artery disease, diabetic foot, and the identification of many other abnormalities.

Typically, HSI instruments are point or slit imagers that scan the object of interest temporally, in order to produce a two-dimensional image, or use optical bandpass filters to scan the scene spectrally. Examples are the Hadamard encoding slit spectrometer [10], HIS imagers using liquid crystal and acousto-optic tunable filters [11], Fourier transform spectrometers [12], spectro-temporal scanners [13], and more recently volume holographic methods [14]. A tunable laser source has also been employed coupled to a custom-built fundus camera, to sweep the working wavelength from 420 to 1000 nm at steps of 2 nm, eliminating the conventional Xenon flash lamp, with images being registered by a 1.3 megapixel charged-coupled (CCD) camera, to fill the spatial-spectral hypercube [15]. All these serial acquisition systems collect only a fraction of the full data cube at a single instant in time and trade off critical imaging parameters, such as image size, speed, resolution, or signal-to-noise ratio [16]. Various new HIS techniques have been developed lately to overcome these problems. Bernhardt utilized an HSI system with rotational spectrotomography to detect all available photons from an object while obtaining enough information to reconstruct the data cube [17]. Johnson et al. [18] used a computed tomographic imaging spectrometer (CTIS) to capture both spatial and spectral information in a single frame without moving parts or narrow-band filters, and with high optical throughput, which is well suited for human retina imaging with constantly moving eyes. CTIS captures the spatial and spectral information of the retina by imaging the scene through a two-dimensional grating disperser which produces multiple, spectrally dispersed images of the retina that are recorded by a focal plane array (FPA). From the captured intensity pattern, computed-tomography algorithms are used to reconstruct the scene into a “cube” of spatial (*x* and *y*) and spectral (wavelength λ) information. The image cube in wavelength space is then reconstructed from a *single* image [18]. The basic CTIS design uses just two lenses and a focal plane detector. The CTIS instrument concept originated in Japan [19] and Russia [20] and has been advanced to maturity by a group at the Jet Propulsion Laboratory in Pasadena [21] and one at the University of Arizona [22,23]. Trade-off problems between imaging acquisition rate and signal throughput in scanning-based techniques also led to the development of image mapping spectroscopy (IMS), [16,24,25] which captures the whole data cube in a single snapshot without compromising image resolution, speed, optical throughput, or intensive post-processing. The IMS is based on the image mapping principle: the device is coupled to the back image port of a traditional retinal imaging camera [26] and the intermediate image at the entrance port is re-imaged onto a custom fabricated image mapper which consists of hundreds of tiny mirror facets that have a two-dimensional tilt [27]. The image mapper cuts the intermediate image into strips and reflects them toward different locations of a CCD camera. Due to differences in the tilt-angle of the mirror facets, blank regions are created between adjacent image strips at the detector plane. The strips of reflected light from the image mapper are further dispersed by means of a prism array, and re-imaged onto their associated blank regions by an array of re-imaging lenses. Thus, each pixel on the CCD camera is encoded with unique spatial and spectral information from the sample. Finally, the hyperspectral datacube (*x,y,λ* ) is calculated by a re-mapping algorithm [27]. The IMS is one of the first real-time, non-scanning techniques [26,28].

In ophthalmology, HIS has been used to detect various retinal abnormalities. Among the most significant ones is the age-related macular-degeneration (AMD), which is a major cause of blindness in the elderly. Its prevalence increases exponentially with every decade after age 50 [29]. Cell protein cytochrome-c has been identified as a key signaling molecule in the degeneration processes and apoptosis. Schweizer et al. [30] developed an HSI system to collect spectroscopic data, which provided information about the oxidative state of cytochrome-c during oxidative stress for detection of AMD. Another group [25] applied CTIS to quantify the macular pigment (MP) in healthy eyes. They successfully recovered the detailed spectral absorption curves for MP in vivo that correspond to physically realistic retinal distributions.

### Retinal oxymetry

The proper functioning of the retina depends on the availability of an adequate amount of oxygen. Therefore, measuring the amount of oxygen present in the retinal vessels is important in order to detect and monitor diseases such as glaucoma and diabetic retinopathy. The main chromophor of blood is hemoglobin, which is a special protein contained in red blood cells (RBC). As light propagates through a blood sample, absorption and scattering take place. The absorption is due to the hemoglobin contained in the red blood cells and scattering is due to the discontinuities of refractive indices between RBCs and the plasma in which they are suspended. The absorption characteristics of blood can be expressed by the extinction coefficients of hemoglobin which can be found into two states: oxygenated (HbO_2_) and deoxygenated (Hb). Generally, blood oxygen saturation is estimated based on the variation of blood spectra with oxygen saturation. There are two primary vascular networks that provide retina with nutrition: the choroid and the retinal vessels. The choroid lies beyond the outer retina, with a capillary bed in contact with the retinal pigment epithelium. Retinal vessels occupy the inner half of the neural retina, extending outward from the optic disc in all directions. As the wavelength of illuminating light changes, light penetrates to different depths throughout the retina in which wavelengths between 530–580 nm illuminate the retinal background and retinal vessels. However, as wavelength increases (λ > 600 nm), light penetrates the retinal vessels and background to reach the choroid at λ > 640 nm [31]. Assuming blood can be spectrally characterized as comprising fully oxygenated hemoglobin (HbO_2_) and deoxygenated hemoglobin (Hb), the oxygen saturation *OS* is defined as:

$$\mathrm{OS}=\frac{C_{\mathrm{Hb}O_{2}}}{C_{\mathrm{Hb}O_{2}}+C_{\mathrm{Hb}}}$$

Where *CHbO*_*2*_ and *CHb* are the molar concentrations of oxygenated and deoxygenated hemoglobin respectively. Several study groups have employed the existing spectroscopic techniques to measure retinal blood oxygen saturation, which involves detecting the difference in light absorption between oxygenated and deoxygenated hemoglobin using multiple wavelength reflectance oximetry. As a result, numerous dual- and multiple wavelength combinations sensitive to oxygen saturation have been utilized in various imaging systems. A good historical summary of such techniques is given in [31].

Modern retinal oxymetry uses hyperspectral imaging methods, to add a topological component to the retinal oxygenation information [15,31-37]. Khoobehi et al. [34] attached a fundus camera to an HSI for monitoring relative spatial changes in retinal oxygen saturation. The integrated system can be adapted to measure and map relative oxygen saturation in retinal structures and the optic nerve head in nonhuman primate eyes. Another team [36] measured the intensities of different wavelengths of light that were transmitted through and reflected out of the arteries, veins, and the areas surrounding these vessels. A hyperspectral fundus imaging camera was used to capture and analyze the spectral absorptions of the vessels. Johnson and co-workers developed a snapshot HSI system with no moving parts or narrow-band filters in order to perform functional mapping of the human retina using chromophore spectra [18]. It was based on the CTIS design, mentioned above. The hemoglobin spectral signatures provided both qualitative and quantitative oxygen saturation maps for monitoring retinal ischemia from either systemic diseases, such as diabetes, or from localized retinal arterial and vascular occlusions, which are the leading causes of untreatable blindness. The results showed a clear distinction between veins, arteries, and the background. Regions within vessel capillaries agreed well with the 30 to 35% oxygen saturation difference expected for healthy veins and arteries. The saturation for most of the background spatial locations in between the capillary regions showed a tendency to be within the 90 to 100% range, which was consistent with the subjects being healthy. This system is capable of acquiring a complete spatial-spectral image cube of 450 to 700 nm with 50 bands in ca. 3 ms and without motion artifacts or pixel misregistration [18].

### Confocal microscopy

**Figure 3:**
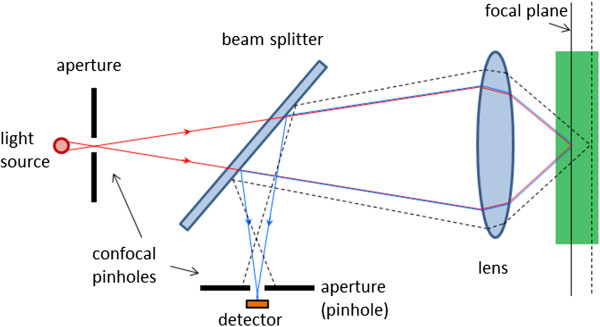
**The principle of confocal microscopy.** Only light reflected by structures very close to the focal plane can be detected.

In order to better understand the material in some of the following sections, we need to introduce the concept of confocal microscopic imaging, which was patented in 1955 by Marvin Minsky [38]. This technique has successfully been utilized in numerous instruments in different areas of science and engineering. A confocal microscope uses point illumination and a pinhole (also called confocal filter) in an optically conjugate plane in front of the detector to eliminate out-of-focus signal (Figure 3). Only light reflected by structures very close to the focal plane can be detected. However, since much of the light returning from the specimen is blocked at the pinhole, the increased resolution comes at the cost of decreased signal intensity, i.e. either a more powerful light source or a long exposure is needed. As only one point in the sample is illuminated and acquired at any given instant, 2D imaging requires scanning over a regular raster in the specimen. The achievable thickness of the focal plane is defined mainly by the wavelength of the used light divided by the numerical aperture(the range of angles over which the system can accept or emit light) of the focusing lens, but also by the optical properties of the specimen. Factors affecting axial (depth) resolution are the objective numerical aperture (NA) and pinhole diameter. Increasing the NA and/or decreasing the diameter of the pinhole will increase the *z*-resolution. The thin optical sectioning makes confocal microscopes particularly good at 3D imaging. By scanning many thin sections through the sample, one can build up a very clean three-dimensional image of the sample. The main advantage of confocal microscopy is the controllable depth of field, suppression of out-of-focus information, and ability to provide optical sections at different depths [39].

### Scanning Laser Ophthalmoscope (SLO)

The first attempt to introduce an ophthalmic imaging technique which would not suffer from the disadvantages of fundus photography was the scanning laser ophthalmoscopy. It was reported first by Webb and co-authors [40,41]. In the scanning laser ophthalmoscope (SLO), a narrow (ca. 1 mm) laser beam of safe intensity traverses the optical axis to a single point (ca. 10 μm diameter) on the retina, resulting in comfort for the patient. The fundus image was produced by scanning the laser over the retina in a raster pattern, detecting the signal from each point scanned, to produce a digital image. Beam deflection was achieved by a combination of two galvanometer scanners – one slow vertical scanner (~60 Hz), and one fast horizontal scanner (~15 kHz). Alternatively, more expensive acousto-optic deflectors can be used [41,42]. Modulation of the scanning beam permits projection of graphics or text in the raster). An avalanche photodetector was initially used, to enhance detector sensitivity. Early SLOs typically provide an output in standard TV format which can be viewed live on a TV monitor and recorded on a videotape, or fed to a digital frame grabber [43,44].

The ability to perform confocal imaging is a major advantage of the SLO [45,46]. The confocal scanning laser ophthalmoscope (cSLO) was developed several years after the SLO as a new version, taking advantage of the principle of confocal microscopy, to achieve high contrast and depth resolution. By moving a confocal aperture between two end points, a number of tomographic slices can be acquired, to extract depth information [47,48].

**Figure 4:**
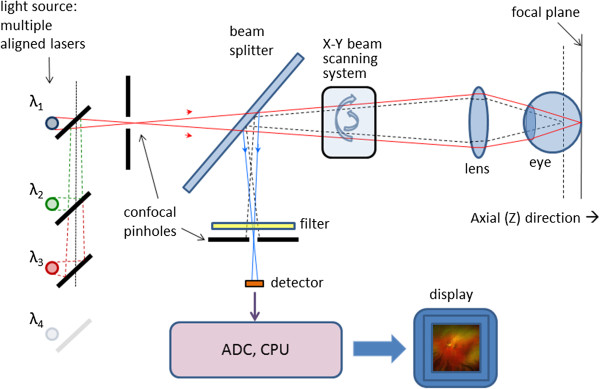
A generalized diagram of a multispectral confocal scanning laser ophthalmoscope (cSLO).

Another important development in scanning laser ophthalmoscopy is the introduction of color, to better match the images produced by fundus photography. Such devices, often called multi-spectral SLOs, use multiple separate lasers of different wavelength in the illumination model, usually made coaxial by means of a set of dichroic combining mirrors. The source lasers are multiplexed, to create interlaced images in a multispectral frame acquisition mode. Multispectral SLOs are usually confocal, and are useful in retinal vessel oximetry, reflectometry, angioscotometry, fundus perimetry etc [44,49-52]. Figure 4 shows a generalized diagram of a multispectral cSLO. The illumination module comprises several separate laser beams combined by dichroic mirrors. The lasers can be of any type, yet more recent designs tend to use diode lasers. The lasers can be multiplexed, or fired simultaneously. The polychromatic beam is made incident on a two-dimensional (X-Y) scanning mirror assembly that displaces it over a square area of several millimeters on the retina. Light reflected from the fundus traverses the incident path in a reverse direction up to a separating beam splitter whereupon a portion is redirected towards the detector. A switchable band-pass optical filter may be placed here to block all other wavelengths but the one of the laser currently turned on, or the wavelength currently being acquired. Recent developments in liquid crystal technology have resulted in the design of electrically tunable tri-color optical filters (red 680 nm, green 550 nm, blue 450 nm) suitable for such applications. Because of laser safety issues [53-55], it is desirable to have only one laser turned on at a time. To obtain information from the lasers needed to build a color image, one can either acquire the monochromatic images consecutively and then merge [56], or generate the color image by pulsing the lasers at such a rate that each point on the imaged area on the retina is illuminated by all colors, one after the other [44]. Latter approach decreases motion artefacts due to eye movements. The receiving path further contains the confocal pinhole and the photodetector, which can be a simple photodiode (covering the wavelengths of interest), or an avalanche photodiode. The pinhole allows passage of light reflected only from the focal plane and blocks scattered light that can blur the image. The result is a focused, high contrast image. The image on the figure is acquired with the Panoramic200 imaging SLO, courtesy of Optos, NA.

The advantages of the cSLO over traditional fundus photography include improved image quality, patient comfort, video capability, and effective imaging of patients who do not dilate well, such as diabetics. The cSLO has been used for detecting biomarkers of diabetic retinopathy [57], as well as age-related macular degeneration [58].

**Figure 5:**
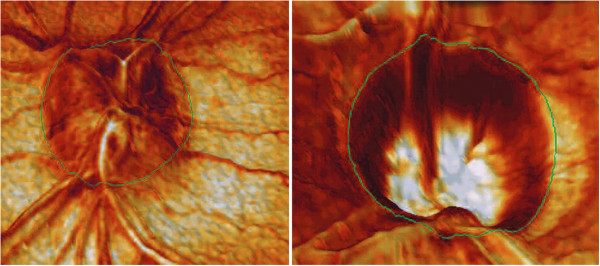
**Three-dimensional view of the retina reconstructed with the Heidelberg Retinal Tomograph (HRT).** Left: 3-D view of optic nerve drusen. Right: 3-D image from a person with advanced glaucoma. Note the depth of the cup, steepness of the walls, and reduced rim tissue. Courtesy of Heidelberg Engineering.

A typical cSLO device is the Heidelberg Retinal Tomograph (HRT) which generates up to 64 transaxial laser scans, to reconstruct a high-resolution 3D image of the fundus using a 670-nm diode laser. A laser light scans the retina in 24 milliseconds, starting above the retinal surface, capturing parallel image sections at increasing depths, which can be combined to create three-dimensional images of the retina. Images are aligned and compared using TruTrack™ technology for both individual examinations and for detecting progression between examinations. The HRT II and HRT III, along with optical coherence tomographs, have become standard instruments for cSLO scanning of the optic nerve head in glaucoma, and is widely being used for imaging the RNFL [59,60]. Figure 5 shows two 3D images of the retina reconstructed with the HRT, courtesy of Heidelberg Engineering.

The left panel is a view of optic nerve drusen. The right panel presents an image from a person with advanced glaucoma. Note the depth of the cup, steepness of the walls, and reduced rim tissue.

SLO image quality is often degraded by effects of involuntary eye movements, especially with patients who cannot fixate properly (i.e. patients with diabetic retinopathy or central scotoma). Since the SLO builds images point-by-point from a flying laser spot, using retinal spatial information from a fixed frame of reference along with retinal eye tracking can significantly improve image quality. This was achieved in a compact *Tracking* SLO (TSLO) with high-speed retinal tracker [61]. The TSLO employs active tracking by placement of a dithered beam originating from a low-power LED onto the fundus and detection and processing of the backscattered reflectance signal by means of a phase-sensitive detection. Feedback is accomplished in real time with a digital signal processor (DSP) thus achieving overall system bandwidth of 1 kHz and significantly enhancing the imaging capabilities of the SLO. Further work in the field of confocal scanning laser ophthalmoscopy has led to the development of relatively simple, low-cost, compact non-adaptive optics, lens-based cSLO designs operating at relatively large field of view (FOV) and throughput, while maintaining resolution adequate for visualizing para-foveal cone photoreceptors and nerve fiber bundles [62].

### Adaptive Optics Scanning Laser Ophthalmoscope (AOSLO)

The scanning laser ophthalmoscopes were further improved by integrating additional technologies into them. The most significant one was adaptive optics (AO), which originated from astronomy [63,64]. With adaptive optics, the performance of optical systems is improved by reducing the effect of wavefront distortions. It is used in astronomical telescopes and laser communication systems, to remove the effects of atmospheric distortion. In retinal imaging systems AO is used to reduce optical aberrations by measuring the distortions in a wavefront and compensating for them with a device that corrects those errors such as a deformable mirror [65-70]. Ocular aberrations are distortions in the wavefront passing through the pupil of the eye. They diminish the quality of the image formed on the retina. Spectacles and contact lenses correct low-order aberrations, such as defocus and astigmatism. With retinal imaging, light returning from the eye is subject to similar wavefront distortions caused by spatial phase nonuniformities, deteriorating the quality of the image and the ability to resolve microscopic retinal structures such as cells and capillaries. In order to achieve microscopic resolution, high-order aberrations, such as coma, spherical aberration, and trefoil, often not stable over time, must also be corrected.

The adaptive optics scanning laser ophthalmoscope (AOSLO) measures ocular aberrations using a wavefront sensor, most commonly the Shack-Hartmann sensor. In a Shack-Hartmann wavefront sensor, the nonuniformities in the wavefront are measured by placing a two-dimensional array of small lenses (lenslets) in a pupil plane conjugate to the eye's pupil, and a CCD chip at the back focal plane of the lenslets. The lenslets cause spots to be focused onto the CCD chip, and the positions of these spots are calculated using a centroiding algorithm. The positions of these spots are compared with the positions of reference spots, and the displacements between the two are used to determine the local curvature of the wavefront—an estimate of the phase nonuniformities causing aberration. Once the local phase errors in the wavefront are known, they can be corrected by placing a phase modulator (wavefront compensator) such as a deformable mirror at yet another plane in the system, conjugate to the eye's pupil. The phase errors can be used to reconstruct the wavefront, which can then be used to control the deformable mirror. AOSLO systems, although usually more complex than the “standard” cSLO, have proven to deliver excellent high-contrast imaging quality at high axial resolution [71-74].

With AO systems, the high magnification necessary to resolve small structures such as photoreceptors are concomitant with smaller fields of 1-2° (ca. 400–500 μm). This requires also image stabilization. An image-based eye-tracking and stimulus delivery method has been implemented into an AOSLO [75-78]. In [74] retinal image was stabilized to 18 μm 90% of the time using a tracking AOSLO. This stabilization was sufficient for cross-correlation techniques to automatically align images. The detection system incorporated selection and positioning of confocal apertures, allowing measurement of images arising from different portions of the double pass retinal point-spread function (PSF).

### Scanning Laser Polarimetry (SLP)

The RNFL is not constant across the retina. It can also change with time – as nerve fibers die with advancing glaucoma, the RNFL becomes thinner. This corresponds to decreased amount of birefringence, which can be detected by a device called scanning laser polarimeter (SLP). The SLP incorporates polarimetry into a scanning laser ophthalmoscope, in order to detect the birefringence of the RNFL. Ellipsometry and polarimetry are often used interchangeably. Strictly speaking, ellipsometry measures the polarization state of light, whereas polarimetry often refers to measuring the angle of rotation caused by retardation when passing polarized light through, or reflecting light by an optically active substance. Birefringence in the retina was first observed by several investigators in the 1970s and early 1980s [79-81]. In the mid-to-late 1980s, human foveal birefringence was measured in vivo with Mueller-matrix ellipsometry [82]. In the early 1990s, the birefringence of the retinal nerve fibers was utilized by Dreher and collaborators [4] to measure the thickness of the nerve fiber layer, again, using a retinal laser ellipsometer.

**Figure 6:**
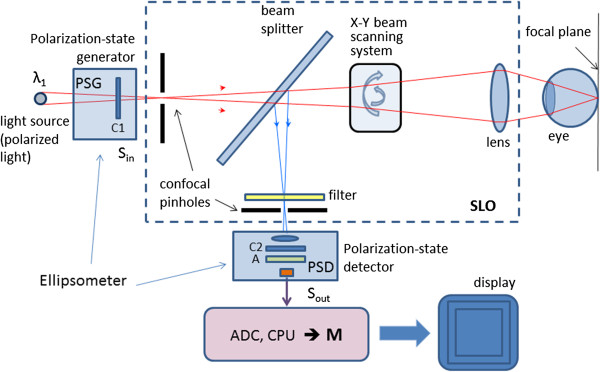
**A simplified general diagram of a scanning laser polarimeter (SLP), consisting of a scanning laser ophthalmoscope (SLO), a polarization-state generator (PSG) and a polarization-state detector (PSD).** The SLP sends a laser beam to the posterior retina and assesses the change in polarization of the reflected beam. This birefringence in the case of the RNFL is caused by neurotubules within the ganglion cell axons.

In the meantime, the theory of Mueller matrix ellipsometry was developed in the late 1970s as a convenient automatic method to measure polarization states and polarization properties of optical media [83,84]. It is well known that the polarization state of light can be described by the Stokes vector **S** = {S_0_, S_1_, S_2_, S_3_}, with S_0_ representing the intensity of the wave, while S_1_, S_2_ and S_3_ are linearly independent and describe fully the polarization state of light. The transmission or reflection properties of an optical medium can be represented by the 4×4 Mueller matrix **M**[85,86]. The change in polarization introduced to a light beam can be described as a multiplication of the Mueller matrix of the polarization-changing structure applied to the Stokes vector of the incident light. Thus, the performance of the birefringent material (called also a retarder) can be described as:

(1) $$S_{\mathrm{out}}=M\times S_{\mathrm{in}}$$

where **S** is the 4-element Stokes vector, and **M** is the 4×4 Mueller matrix, whose values are functions of the azimuth *θ* and the retardance *δ* of the corresponding retarder. This also means that the birefringence represented by the Mueller matrix **M** can be measured by giving different values to the input Stokes vector **S**_in_ and measuring every time the output vector **S**_out_, then solving a set of equations, to obtain **M**. Consequently, the Mueller matrix elipsometer has two necessary components: the polarization-state generator (PSG) containing a linear retarder (compensator) C1, and a polarization-state detector (PSD) containing a second retarder (compensator) C2 and a linear analyzer (polarizer) A (Figure 6) [83]. It has been shown [84] that if the PSD consists of a quarter-wave plate rotating at speed ω and the PSD contains a quarter-wave plate rotating synchronously at a speed of 5ω, and the light flux is linearly detected, then a periodic signal.

(2) $$F=a_{0}+\sum_{n=1}^{12}\left(a_{n}\cos\left(n\omega_{f}t\right)+b_{n}\sin\left(n\omega_{f}t\right)\right)$$

is generated, with fundamental frequency ω_f_ = 2ω. From the Fourier amplitudes a_0_, a_n_, b_0_, b_n_, which can be measured by performing a discrete Fourier transform of the signal, the 16 elements of the Mueller matrix can directly be determined [84]. This principle was used in the first SLP for measuring the thickness of the RNFL [3,4]. A simplified diagram of a SLP is shown in Figure 6. The SLP sends a laser beam to the posterior retina and assesses the change in polarization (also called retardation) of the reflected beam. This birefringence in the case of the RNFL is caused by neurotubules within the ganglion cell axons.

**Figure 7:**
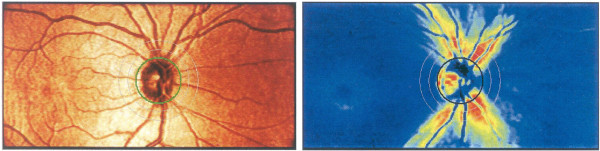
**Images generated by the GDx VCC.** Left: the reflectance image, displayed as a color map; Right: the retardation map converted to color-coded RNFL thickness, with thinner regions displayed in blue or green, while thicker regions are displayed in yellow or red. Courtesy of Carl Zeiss Meditec, Inc.

One such SLP device, developed specially for the purpose of identifying glaucoma, is the GDx nerve fiber analyzer (developed by Laser Diagnostic Technologies and marketed later by Carl Zeiss Meditec) [87]. The laser scanning is based on the principle of the cSLO. The device generates a high-resolution image of 2565×256 pixels created by measuring the retardation of the laser scan at each location. Thus, RNFL thickness maps are generated representing the likelihood of glaucomatous RNFL loss. For each measurement, the GDx generates two images: a reflection image and a retardation image. The reflectance image is generated from the light reflected directly back from the surface of the retina, and is displayed as the Fundus Image on the device printouts. The retardation image is the map of retardation values and is converted into RNFL thickness based on a conversion factor of 0.67 nm/μm [5]. Figure 7 shows two images generated by the GDxVCC, courtesy of Carl Zeiss Meditec. The left image is the reflectance image, displayed as a color map. The right image is the retardation map converted to color-coded RNFL thickness, with thinner regions displayed in blue or green, while thicker regions are displayed in yellow or red [87].

**Figure 8:**
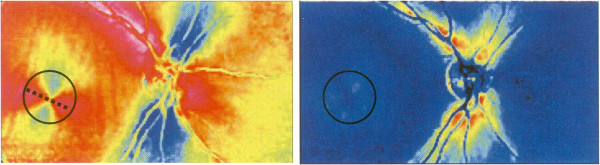
**Compensating the anterior segment birefringence in the GDx VCC (macula and optic nerve head).** Left: The uncompensated retardation image, which includes the retardation from the cornea, lens and RNFL. The retardation profile in the macula is due to the cornea, lens, and macula itself (Henle fiber layer). The axis of birefringence is shown as a dashed line. Once the axis and the magnitude values are known, the variable compensator VCC can be set to compensate for the anterior segment birefringence. Right: The resulting compensated image. The retardation profile in the macula is now uniform due to compensation.

It should be pointed out that in addition to the RNFL, also the cornea and the eye lens cause birefringence, commonly referred to as *anterior segment retardation*. Several methods have been proposed for compensation of anterior segment birefringence in scanning laser polarimetry [88-93]. At first, a fixed corneal compensator (FCC) was used. It was a retarder of fixed magnitude (60 nm) and fixed fast axis orientation (15° nasally down). Later, a variable corneal compensator (VCC) was introduced to individually compensate corneal retardance in terms of retardance magnitude and azimuth [87,88,94]. This technique was implemented in the GDxVCC: first the uncompensated image is acquired, which includes the retardation from the cornea, lens and RNFL. The macular region (containing the fovea) of this image is then analyzed to determine the axis and magnitude of the anterior segment birefringence [88]. The macular region birefringence is uniform and symmetric due to the radial distribution of the Henle fiber layer, which is made up of parallel photoreceptor neuronal processes that are radial and horizontal to the retinal surface in the center of the fovea. However, in uncompensated scans, a non-uniform retardation pattern is present in the macula due to the birefringence from the anterior segment (Figure 8). The axis orientation (azimuth) and magnitude values from the anterior segment can be computed by analyzing the non-uniform retardation profile around the macula. The axis of the anterior segment is determined by the orientation of the “bow-tie” birefringent pattern, and the magnitude is calculated by analyzing the circular profile of the birefringence in the macula. Once the axis and the magnitude values are known, the variable compensator VCC can be set to compensate for the anterior segment birefringence [87,88]. Later, an enhanced corneal compensation algorithm (ECC) was introduced by Zeiss to the GDx technology. With it, a known large birefringence bias is introduced into the measurement beam path to shift the measurement of total retardation into a higher value region. The birefringence bias is determined from the macular region of each measurement, and then, point-by-point, removed mathematically, to yield true RNFL retardation [95]. In another study, the authors suggested an algorithm for calculating birefringence that uses *large* areas of the macula available in the images, to achieve better signal-to-noise ratio. The uncertainty of the calculated retardance was estimated, and an appropriate averaging strategy to reduce uncertainty was demonstrated [90].

**Figure 9:**
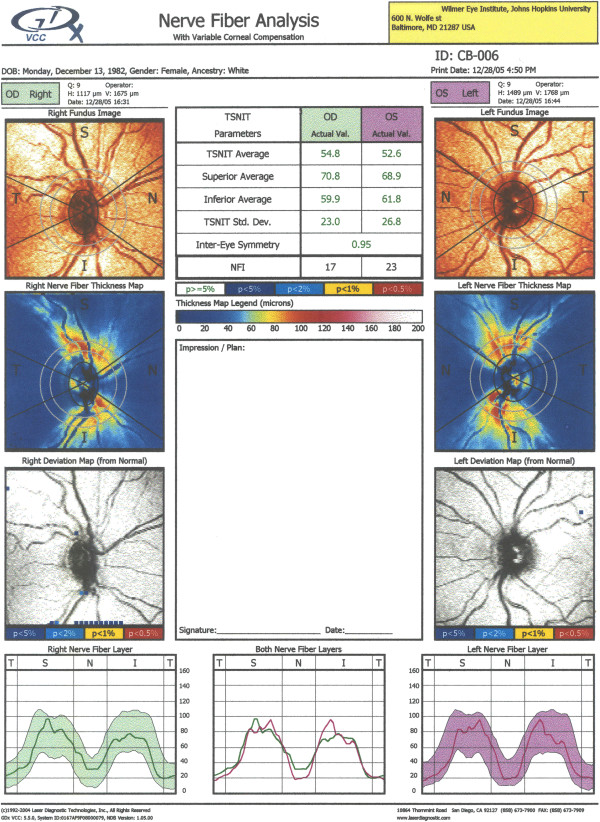
**GDxVCC – exam printout of a normal subject.** Key elements: a) the fundus image (top row; useful to check for image quality); b) the thickness map (second row) showing the thickness of the RNFL on a scale of 0 (dark blue) to 120 μm (red), with yellow-red colors for a healthy eye; c) the deviation maps (third row) revealing the location and severity of RNFL loss over the thickness map in serial comparison of thickness maps; d) the Temporal-Superior-Nasal-Inferior-Temporal (TSNIT) maps (bottom row), displaying the thickness values along the calculation circle starting temporally and moving superiorly, nasally, inferiorly and ending temporally, along with the shaded areas representing the 95% normal range for the patient’s particular age. The printout also includes parameters, such as the TSNIT average, Superior average, Inferior average, TSNIT standard deviation and Inter-eye Symmetry.

Figure 9 shows a single exam printout from the GDxVCC, taken at the author’s institution (Wilmer Eye Institute at Johns Hopkins University School of Medicine). Its key elements are: a) the fundus image (top row; useful to check for image quality); b) the thickness map (second row) showing the thickness of the RNFL on a scale of 0 (dark blue) to 120 μm (red), with yellow-red colors for a healthy eye (the pink and white areas are present only in uncompensated cornea scans); c) the deviation maps (third row) revealing the location and severity of RNFL loss over the thickness map in serial comparison of thickness maps; d) the Temporal-Superior-Nasal-Inferior-Temporal (TSNIT) maps (bottom row), displaying the thickness values along the calculation circle starting temporally and moving superiorly, nasally, inferiorly and ending temporally, along with the shaded areas representing the 95% normal range for the patient’s particular age. The printout also includes parameters, such as the TSNIT average, Superior average, Inferior average, TSNIT standard deviation and Inter-eye Symmetry (Figure 9) [87].

It should be noted that with respect to RNFL thickness measurements, other modalities, such as cSLO and optical coherence tomography have proven to be successful alternatives to SLP.

### Retinal Birefringence Scanning (RBS)

**Figure 10:**
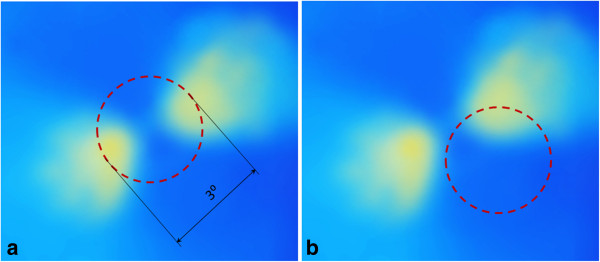
**Retinal Birefringence Scanning (RBS).** A birefringence image of the fovea with the scanning circle (3° of visual angle). The circle can be centered on the fovea during central fixation as in **(a)**, or to the side of the center of the fovea during para-central fixation – as in **(b)**.

**Figure 11:**
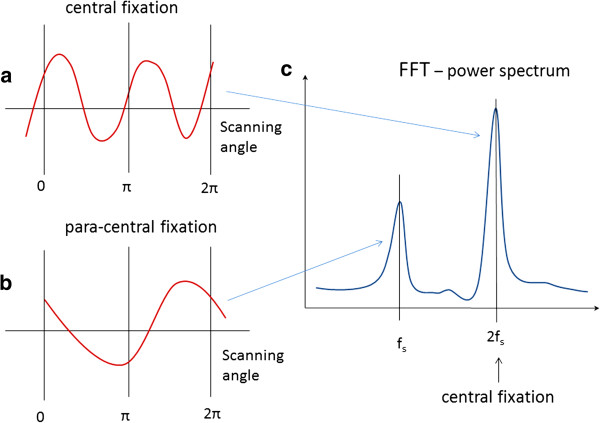
**Signals produced by RBS: a) during central fixation and b) during para-central fixation.** The power spectrum **(c)** contains two peaks – one at 2f_s_, characteristic of central fixation, and one at f_s_, characteristic of para-central fixation.

A special type of polarimetry called Retinal Birefringence Scanning (RBS) was developed in the author’s laboratory, mainly for detection of central fixation and eye alignment - important for identifying risk factors for amblyopia (“lazy eye”) [96-100] RBS is a technique that uses the polarization changes in the light returning from the eye to detect the projection into space of the array of Henle fibers around the fovea. In RBS, polarized near-infrared light is directed onto the retina in a circular scan, with a fixation point in the center, and the polarization-related changes in the light retro-reflected from the ocular fundus are analyzed by means of differential polarization detection. Due to the radial arrangement of the birefringent Henle fibers, a bow-tie pattern of polarization states results, centered on the fovea, with maximum and minimum areas of the polarization cross approximately 1.5° from the center of the fovea. Figure 10(a) and (b) shows a birefringence image of the fovea taken with the GDxVCC before anterior segment compensation (courtesy of Carl Zeiss Meditec). The red dashed circle of diameter of 3° of visual angle represents the scanning path, which can be centered on the fovea (during central fixation as in Figure 10(a), or to the side of the center of the fovea (during para-central fixation – as in Figure 10(b). During central fixation, the concentric circle of light falls entirely on the radial array of Henle fibers, and generates a characteristic birefringence signal which is *twice* the scanning frequency f_s_ (two peaks and two dips per scan), as shown in Figure 11(a). This leads to the appearance of a peak at 2f_s_ in the power spectrum, shown in Figure 11(c). During paracentral fixation, the scan is decentered with respect to the center of the fovea, and the orientation of the radially arranged nerve fibers changes only once during each single scan, resulting in a main frequency component equal to the scanning frequency f_s_. Thus, spectral analysis of the back-reflected signal from the foveal region allows detection of central fixation for that particular eye.

**Figure 12:**
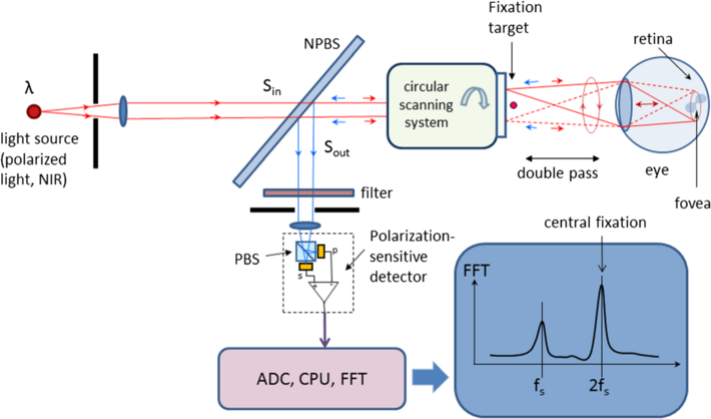
**Basic design of an RBS system.** The light retro-reflected from the retina is of changed polarization, which is measured by a polarization-sensitive detector.

Figure 12 shows the basic design of a RBS system. A “scanning” near-infrared source of polarized light, typically a low-power laser diode, produces linearly vertically polarized light at wavelength λ (λ =785…830 nm), which after collimation arrives at a non-polarizing beam splitter (NPBS). Half of the light continues towards a circular scanning system, which can consist of two plane mirrors. The incoming beam is converted into a circular scan, subtending an angle of approximately 3° at the subject’s eye. By the eye’s own optics, the beam is focused onto the retina, with the eye fixating on the image of small light target, appearing in the center of the scanning circle. The light follows the same path back out of the eye after being reflected from the ocular fundus. The NPBS redirects the retroreflected light to a bandpass filter and a polarization-sensitive photodetector. The polarizing beam splitter PBS separates the light of changed polarization into two orthogonal components (s- and p-) and each one them is detected by a separate photodetector. The vertical polarization component (s) is transmitted by the polarizing beam splitter (PBS) towards the first photodetector, whereas the horizontal component (p) is reflected towards the second photodetector. The second component of the Stokes vector, S_1_, is obtained by building the difference of the two polarization components [85,86]. The difference signal is amplified, digitized and spectrally analyzed in software. Fast-changing and short-lasting spectral components indicative of intermittent central fixation can be detected using time-frequency methods, as described in [101].

In a binocular configuration of the above described system, by analyzing frequencies in the RBS signal from each eye, the goodness of binocular eye alignment can be measured, and thus strabismus (as a risk factor for amblyopia) can be detected. In a number of studies and in several prototypes, RBS has demonstrated reliable non-invasive detection of foveal fixation, as well as detection of eye misalignment [99,102-105]. Also, a non-moving-part design was developed [106], and its ability to perform eye-tracking after calibration was successfully tested [107]. A more recent study has led to the optimization of the parameters of the optical components used in RBS and to improvement of the signal-to-noise ratio across a wide population [108]. RBS has also been shown to work for biometric purposes by identifying the position of the retinal blood vessels around the optic nerve [109], and for identification of Attention Deficit and Hyperactivity Disorder (ADHD) by assessing the ability of test subjects to stay fixated on a target [100].

### Optical Coherence Tomography (OCT)

Optical Coherence Tomography (OCT) is an imaging technique that utilizes the interferometry. The interferometer invented by Michelson sent a beam of light through a half-silvered mirror (beam splitter) splitting the beam into two paths. After leaving the beam splitter, the beams travelled out to the ends of long arms where they were reflected into the middle of small mirrors, and were then recombined in an eye piece, producing a pattern of interference. If the two optical paths differ by a whole number of wavelengths, the interference is constructive, delivering a strong signal at the detector. If they differ by a whole number and a half wavelengths (odd number of half-wavelengths), the interference is destructive and the detected signal is weak.

It can be shown [110] that the intensity measured at the photodetector of a low-coherene interferometer is a sum of three components – the backscattered intensities received respectively from the sample and reference arm, and the interference signal that carries the information about the structure of the sample, and depends on the optical path delay between the sample and the reference arm:

(3) Idτ=Is+Ir+2IsIrReVmcτ

where

(4) $$V_{\mathrm{mc}}\left(\tau \right)=\frac{\langle E_{s}\left(t\right)E_{r}^{*}\left(t+\tau \right)\rangle }{\sqrt{I_{s}I_{r}}}$$

and *τ* is the time delay corresponding to the round-trip optical path length difference between the two arms:

(5) $$\tau =\frac{\mathrm{\Delta L}}{c}=\frac{L_{s}-L_{r}}{c}=\frac{2n\left(l_{s}-l_{r}\right)}{c}$$

with *c* being the speed of light, *n* - the refractive index of the medium, and *l*_*s*_ and *l*_*r*_ - the geometric lengths of the two arms. The normalized mutual coherence function *V*_*mc*_*(τ)* in the above equation is a measure of the degree to which the temporal and spatial characteristics of the source and reference arm match. Since a temporal coherence function is actually the Fourier transform of the power spectral density *S(k)* of the light source (Wiener-Khinchin theorem), the above equations can be rewritten to [110-112]:

(6) IdΔL=Is+Ir+2IsIr ℑ Skcosk0ΔL

where *k*_*0*_ *= 2π/λ*_*0*_ is the average wave number and the relation *λ*_0_ = *c/f*_*0*_ is used to transform from the time domain to the path domain [110].

**Figure 13:**
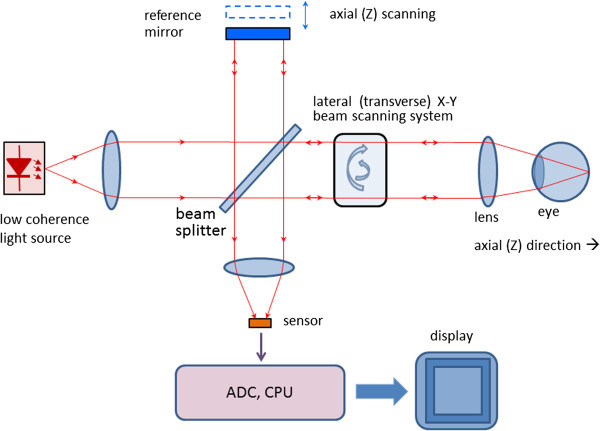
**Typical optical setup of an OCT system containing a moving reference mirror (Time-Domain OCT).** Free space design with no fiber optics.

With OCT, as with the classical Michelson interferometer, light is split into two arms – a sample arm scanning the retina, and a reference arm, which is typically a mirror. After reflection (respectively from the sample and from the reference mirror) light is recombined and directed to the sensor, which can be a simple photodetector, or a camera. Figure 13 shows a typical optical setup of an OCT system containing a moving reference mirror. Systems containing a movable mirror are also called time-domain (TD) OCT systems. A measurement beam emitted by the light source is reflected or backscattered from the object (the retina) with different delay times, depending on the optical properties of the layers comprising the object. A longitudinal (axial) profile of reflectivity versus depth is obtained by translating the reference mirror, thus changing the path length in the reference arm. For each point on the retina, the magnitude of the intensity of the resulting interference fringes is recorded for each position of the reference mirror, i.e. for each depth. In order to extract the depth-signal carrying component, the detection electronics usually contains three main circuits: a) a transimpedance amplifier, b) a band-pass filter centered at the Doppler frequency defined as f_d_ = 2*ν* / *λ*_*0*_ (*ν*: speed of the moving mirror; *λ*_*0*_: the central wavelength of the light source), and c) an amplitude demodulator to extract the envelope of the interferometric signal [113].

**Figure 14:**
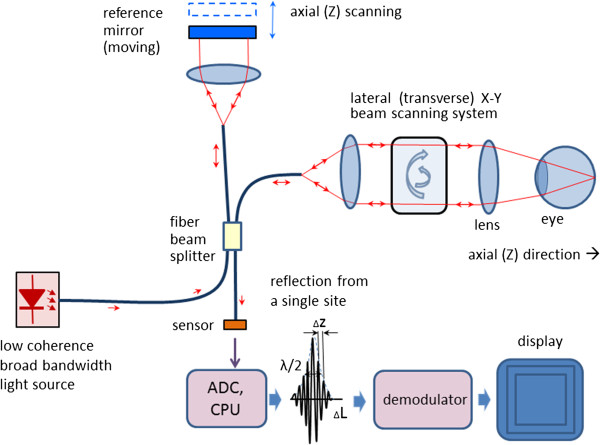
A generalized design of a fiber-based Time-Domain OCT system.

Scanning the light beam on the retina enables non-invasive cross-sectional imaging with micrometer resolution. OCT is based on low coherence interferometry [114-117]. In conventional interferometry with long coherence length, which is the case with laser interferometry, interference occurs over a long distance (meters). In OCT, low coherence light is used. A low coherence light source consists of a finite bandwidth of frequencies rather than just a single frequency. Thanks to the use of broadband light sources (emitting over a broad range of frequencies), this interference is shortened to a distance of micrometers. Broad bandwidth can be produced by superluminescent light emitting diodes (SLDs) or lasers emitting in extremely short pulses (femtosecond lasers). With no lateral X-Y scanning, the information from only one point on the retina is read, at a depth defined by the position of the reference mirror. Lateral (transverse) scanning provides a 2D image for the particular depth chosen. In some designs, instead of X-Y scanning, a camera functioning as a two-dimensional detector array was used as a sensor (full-field OCT optical setup). There are two types of designs that use a moving reference mirror – a free-space and a fiber-based design. A free space design (as in Figure 13) can provide very high resolution images by using custom designed lenses, compensating components in the reference arms, and dynamic focusing to prevent loss of contrast [118]. Instead of dynamic focusing, the more popular fiber-based systems reduce the effects of transversal (lateral) resolution loss by acquiring and subsequently fusing multiple tomograms obtained at different depths at the same transverse location [110,119-122]. Figure 14 shows a generalized fiber-based TD OCT system.

OCT typically employs near-infrared (NIR) light. The use of relatively long wavelength allows light to penetrate deeper into the scattering medium. Confocal microscopy, as used in cSLOs, typically penetrates less deeply into the retina. The transverse resolution for optical coherence tomography is the same as for conventional microscopy, being determined by the focusing of the optical beam. The minimum size to which an optical beam can be focused is inversely proportional to the numerical aperture of the angle focus or the beam [110,123]:

(7) $$\mathrm{\Delta x}=\frac{4\lambda }{\pi }\left(\frac{f}{d}\right)$$

where λ is the wavelength, *d* is the spot size on the objective lens, and *f* is the focal length. High transverse resolution can be achieved by using a large numerical aperture and focusing the beam to a small spot size. In addition, the transverse resolution is related to the depth of focus or the confocal parameter *b*, which is two times the Rayleigh range *z*_*R*_:

(8) $$b=2z_{R}=\frac{\pi \;\Delta x^{2}}{2\lambda }$$

With other words, increasing the transverse resolution produces a decrease in the depth of focus. The signal-to-noise ratio (*SNR*) is given by the expression [123]:

(9) $$\mathrm{SNR}=10\;\log\left(\frac{\mathrm{\eta P}}{2\mathrm{h\nu NEB}}\right)$$

where *η* is the quanum efficiency of the detector, *hν* is the photon energy, *P* is the signal power, and *NEB* is the noise equivalent bandwidth of the electronic filter used to demodulate the signal. The axial resolution of OCT is primarily determined by the bandwidth of the low-coherence light source used for imaging. In this aspect, OCT is different from cSLO, where the depth of focus can be limited by the numerical aperture of the pupil of the eye. For a source of Gaussian spectral distribution, the axial resolution, Δz, is

(10) $$\mathrm{\Delta z}=\frac{2\ln\left(2\right)\lambda_{0}^{2}}{\pi \Delta \lambda }$$

where Δλ is the full width at half maximum (FWHM) wavelength range of the light source, and λ_0_ is the center wavelength [113]. Commercial “standard-resolution” OCT instruments use superluminescent diodes (SLD) emitting light centered at 830 nm and 20–30 nm bandwidth, thus resulting in a ~10 μm axial resolution in the retina [124]. Ultrahigh-resolution OCT imaging (UHR OCT) [125,126] achieves better axial resoulution of 2–3 μm thereby enabling visualization of intraretinal structures. This advance was first demonstrated using ultrabroad-bandwidth, solid state femtosecond Titanium:sapphire lasers [127,128] instead of the traditional SLD. Ti:sapphire lasers are capable of providing FWHM of 140–160 nm and in some cases over 250 nm. Further, a frequency-doubled Nd:YVO_4_, 1.8 W laser (Excel, Laser Quantum) was reportedly integrated into the resonator layout, and a prototype of a prismless Ti:sapphire laser of 260 nm bandwidth at FWHM, 6.5 femtosecond pulse duration was developed, for a wavelength range of 640–950 nm [129]. Femtosecond laser technology achieved unprecedented resolution, but is expensive, being suitable mainly for fundamental research. More recently, cost-effective, broad-bandwidth SLD sources have been developed that approach resolutions achieved by femtosecond lasers [130-133]. They comprise multiplexed SLDs consisting of two or three spectrally displaced SLDs, combined to synthesize a broad spectrum. With very wide-spectrum sources emitting over nearly 100 nm wavelength range, OCT has achieved sub-micrometer resolution. Despite the disadvantage of spectrally modulated emission spectra producing sidelobes in the coherence function and image artifacts, multiplexed SLDs are the light source of choice for many commercial instruments, providing 5–8 μm axial resolution [124].

**Figure 15:**
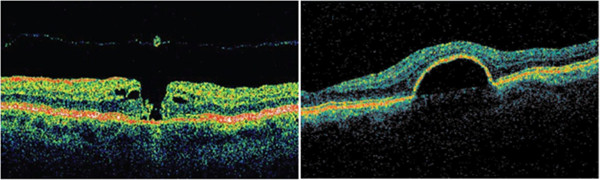
**Pathology examples detected with the TD OCT instrument STRATUS OCT™, courtesy of Carl Zeiss Meditec.** The left panel shows a macular hole with posterior vitreous detachment. The right panel presents pigment epithelial detachment. The structures of the retina are color-coded.

Figure 15 shows pathology examples detected with the TD OCT instrument STRATUS OCT™, courtesy of Carl Zeiss Meditec. The left panel shows a macular hole with posterior vitreous detachment. The right panel presents pigment epithelial detachment. The structures of the retina are color-coded.

### Optical coherence tomography in the Fourier domain (FD OCT, spectral radar, spectral domain OCT)

It can be shown that the cross spectral density function of two waves (in this case the reference and the sample wave) can be obtained as the Fourier transform of the cross-correlation function [110]:

(11) Sijk= ℑ rijΔL

where *k = 2π/λ*_*0*_ is the wave number, *r*_*ij*_*(ΔL)* are the cross-correlation functions of the two waves, *r*_*ij*_*(ΔL)* = c*τ, τ* being the time delay corresponding to the round-trip optical path length difference between the two arms [116]. The amplitude of the spectrum of the backscattered light*, I(k)*, can be measured for different wavenumbers *k* using a spectrometer. The inverse Fourier transform of the measured spectral intensity gives theoretically the same signal as obtained by low coherence interferometry, providing a function of the depth for each point, without a moving reference mirror [110,134,135]:

(12) $$s_{\mathrm{ij}}\left(z\right)={ℑ}^{-1}\left\{r_{\mathrm{ij}}\left(\mathrm{\Delta L}\right)\right\}={ℑ}^{-1}\left\{I\left(k\right)\right\}$$

In fact, similar to (6), the total interference spectrum *I(k)* for a scatterer at a distance *z* can be calculated as [110]:

(13) $$\begin{array}{ll} {ℑ}^{-1}\left\{I\left(k\right)\right\} & ={ℑ}^{-1}\left\{S\left(k\right)\right\}⊗\left\{\left[\delta \left(z\right)\right]+0.5\hat{a}\left(z\right)+0.125H\left[\hat{a}\;\left(z\right)\right]\right\}\\ & =\;A⊗\left(B+C+D\right) \end{array}$$

where *S(k)* is the spectrum of the source. The useful signal C (the middle convolution term) is the scattering amplitude *a(z)*, i.e. the strength of the scattering versus the depth of the sample. The first convolution is the Fourier transformation of the source spectrum located at *z = 0*, and the last convolution stands for the autocorrelation terms, describing the mutual interference of the scattered elementary waves [110].

**Figure 16:**
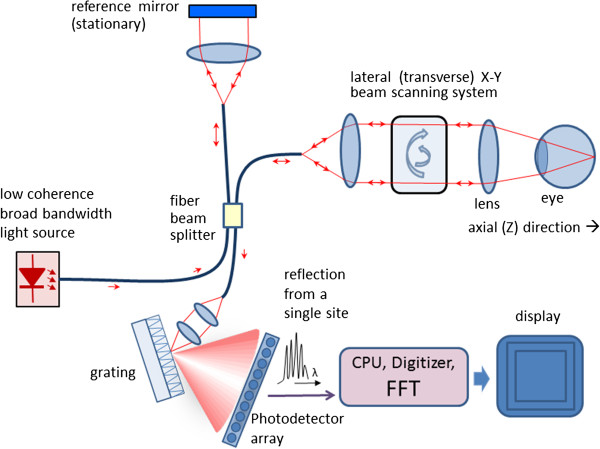
**A typical fiber-optic implementation of the Fourier domain OCT (FD OCT).** The slow mechanical depth scan is replaced by a spectral measurement consisting of diffraction grating and photodetector array.

Thus, compared to TD OCT, with FD OCT only the transversal scanning procedure remains. Figure 16 shows a typical fiber-optic implementation of the Fourier domain OCT. Similar to TD OCT, a broad bandwidth source is used. In contrast to TD OCT, the slow mechanical depth scan is replaced by a spectral measurement consisting of diffraction grating and photodetector array (here a CCD). The signal is measured in the spectral domain and then the Fourier transform delivers the scattering profile in the spatial domain. The interference spectrum *I(k)* for a single scatterer at a certain distance from the reference plane z_1_ is a cosine function multiplied by the source spectrum *S(k)*. The Fourier transform delivers the location of the peak at that frequency that corresponds to the scatterer location. With FD OCT, the measurable axial range is limited by the resolution of the spectrometer. It has been shown [110] that the maximum resolvable depth is

(14) $$Z_{\max}=\frac{\lambda_{0}^{2}}{4n\left(\mathrm{d\lambda}\right)}$$

where dλ denotes the wavelength sampling interval of the spectrometer.

**Figure 17:**
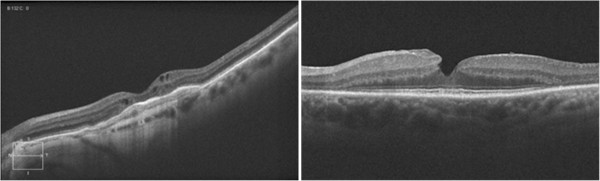
**Pathology examples detected with the CIRRUS HD-OCT™ FD OCT, courtesy of Carl Zeiss Meditec.** The left panel shows age-related macular degeneration. The right panel presents a lamellar macular hole.

**Figure 18:**
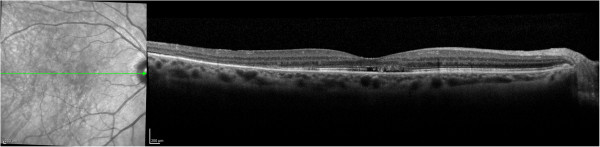
**Photoreceptor disruption of the retina (right), observed in a section marked with a green line on the transversal image (left).** The image was obtained with the SPECTRALIS® FD OCT, courtesy of Heidelberg Engineering.

Figure 17 shows pathology examples detected with the CIRRUS HD-OCT™ FD OCT, courtesy of Carl Zeisss Meditec. The left panel shows age-related macular degeneration. The right panel presents a lamellar macular hole. Figure 18 shows photoreceptor disruption of the retina (right), observed in a section marked with a green line on the transversal image (left). The image was obtained with the SPECTRALIS® FD OCT from Heidelberg Engineering. This instrument has enhanced the role of FD OCT by integrating it with a cSLO. Courtesy of Heidelberg Engineering.

### Swept Source Optical Coherence Tomography (SS OCT, Wavelength Tuning)

**Figure 19:**
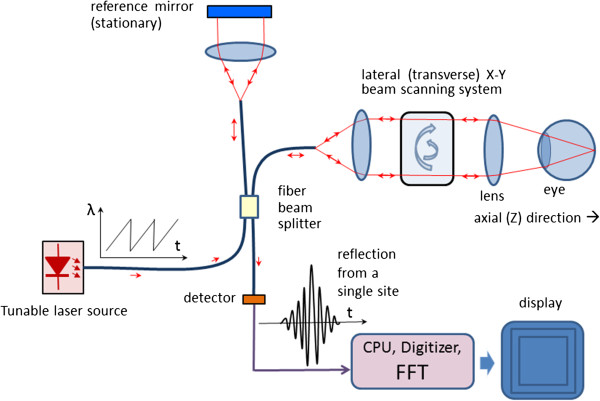
A Swept Source OCT system – typical design.

In Swept Source OCT (Figure 19) the wavelength-dependent intensity data are not acquired simultaneously by using a broadband light source and a spectrometer. Instead, the wavelength of the source is being tuned, and a single photodetector is used, recording wavelengths sequentially [136]. The light intensity at the photodetector at wavelength λ of the tunable laser can be calculated as [137]:

(15) $$I=I_{s}+I_{r}+2\sqrt{I_{s}I_{r}}\cos\left(2\pi \Delta \Phi \right)$$

where *I*_*s*_ and *I*_*r*_are the intensities reflected from the sample and the reference arm, respectively, and ΔΦ is the phase difference between the two beams [110]:

(16) $$\Delta \Phi =2\frac{L}{\lambda }=2L\frac{k}{2\pi }$$

with *k* being the wavenumber corresponding to wavelength λ. The phase difference ΔΦ changes with the wavenumber, causing the intensity at the photodetector to change with a frequency [110]:

(17) $$f=\frac{d\Delta \Phi }{\mathrm{dt}}=\frac{d\Delta \Phi }{\mathrm{dk}}\frac{\mathrm{dk}}{\mathrm{dt}}=\frac{L}{\pi }\frac{\mathrm{dk}}{\mathrm{dt}}$$

The above equation shows that the signal frequency at the detector is directly proportional to the tuning rate of the wavenumber *dk/dt* and the path difference *L*. With a constant *dk/dt* (wavelength λ being a ramp), *L* can be calculated by means of Fourier transform of the time-dependent intensity recorded at the photodetector. Fourier-transforming the time-dependent beat signal yields the sample depth structure. With other words, the magnitude of the beat signal defines the amplitude reflectance while the beat frequency defines the depth position of light scattering sites in the sample [110].

### Polarisation Sensitive Optical Coherence Tomography (PS OCT)

Originally, the emphasis of OCT has been the reconstruction of 2D maps of changes of tissue reflectivity, with depth information. However, in 1992 Hee et al. [138] reported the first OCT system capable of measuring also changes in the polarization state of light (birefringence). In 1997, the first polarization-sensitive (PS) images of biological tissue (bovine tendon) were presented, examining also the effect of thermal damage on collagen birefringence [139]. A further theoretical contribution to the determination of depth-resolved Stokes parameters of backscattered light using PS OCT was made two years later by the same authors [140]. Thus, PS OCT became a functional extension that takes advantage of the additional polarization information carried by the reflected light. In the meantime it had become known that several ocular structures possess birefringent properties. In the retina these are the RNFL around the optic disc [4], which can help in the diagnostics of glaucoma [141], and the Henle fiber layer around the fovea [1], which can be used for detection of macular defects. As reported in [142], the optic nerve head is surrounded by the birefringent sclera rim, which may be used as a landmark in studies of optic disc anatomy. In addition, a polarization scrambling layer is located near the retinal pigment epithelium (RPE) which may become useful in the diagnostics of age-related macular degeneration (AMD) [143]. The main advantage of PS OCT is the enhanced contrast and specificity in identifying structures in OCT images by detecting induced changes in the polarization state of light reflected from the sample. Moreover, changes in birefringence may indicate changes in functionality, structure or viability of tissues [144].

Birefringence changes the polarization state of light by a difference (Δn) in the refractive index for light polarized along, and perpendicular to the optic axis of a material. The difference in refractive index introduces a phase retardation δ between orthogonal light components that is proportional to the distance traveled through the birefringent medium [144]:

(18) $$f=\frac{d\Delta \Phi }{\mathrm{dt}}=\frac{d\Delta \Phi }{\mathrm{dk}}\frac{\mathrm{dk}}{\mathrm{dt}}=\frac{L}{\pi }\frac{\mathrm{dk}}{\mathrm{dt}}$$

**Figure 20:**
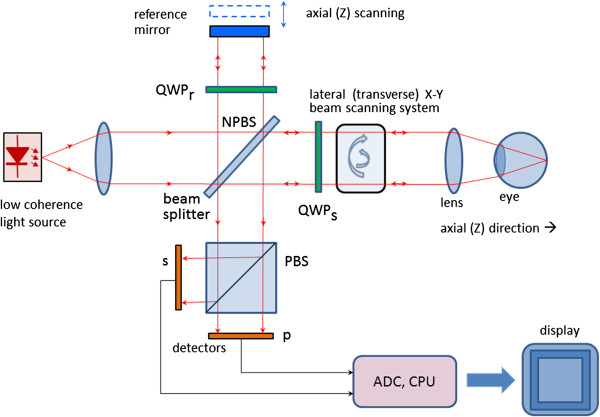
A simplified configuration of a Polarization Sensitive OCT (time-domain).

A simplified configuration of a PS OCT (time-domain) is shown in Figure 20. It is based on early open-air designs [138,140,144,145]. Linearly polarized light (produced by either a laser diode, or a superluminescent diode and a polarizer) is split into reference and sample arm by a non-polarizing beam splitter (NPBS). Light in the reference arm passes through a zero-order quarter-wave plate (QWP_r_) with its slow-axis oriented at 22.5° to the incident horizontal polarization. After reflection from the reference mirror, the light is returned through QWP_r_ , now linearly polarized at 45°, providing equal reference beam power in the two orthogonal directions (vertical and horizontal). Light in the sample arm passes through another quarter-wave plate, (QWP_s_) oriented at 45° to the incident horizontal polarization and through focusing optics, producing circularly polarized light incident on the sample. Light reflected from the sample has generally elliptical polarization, determined by the birefringence of the sample. The reflected light passes through the QWP_s_ again. After recombination in the detection arm, the light is split into its horizontal (p) and vertical (s) linear polarization components by a polarizing beam splitter PBS, and is then measured by corresponding detectors. The two photodetector signals are demodulated separately, to produce a two-channel scan of reflectivity versus distance. Buy using a PBS and quarter-wave plates, and detecting in two orthogonal linear polarization modes, this design is made sensitive to phase retardation and measurements are independent of sample axis rotation in the plane perpendicular to the sample beam [138].

Several groups have reported also fiber-based PS-OCT systems [146-148]. Compared to open-air systems, fiber-based PS-OCT are easier to construct. Yet, in a fiber-based system, maintaining the polarization state in the fiber is a challenge, because of stress in the fibers and a non-circular shape of the fiber core. Further developments include Spectral Domain PS OCT where, just as in standard FD OCT, the reference mirror is stationary and the photodetectors (now a pair) are replaced by a pair of spectrometers. This led to a significant increase of speed [149,150]. More recently, an even faster, Swept Source PS OCT was reported [151] achieving a 350 kHz A-scan rate.

It should be noted that also the cornea is birefringent. This means that a beam probing the retina, be it initially of circular or linear polarization, will have elliptical polarization after passing through the cornea. A similar problem arises in scanning laser polarimetry, discussed earlier, where using a variable retarder helped compensate individual corneal birefringence [152]. An interesting approach was taken by Pircher et al. [147] who report a software-based corneal birefringence compensation that uses the polarization state of light backscattered at the retinal surface to measure corneal birefringence (in terms of retardation and axis orientation) and then compensate corneal birefringence numerically.

An international group [153] recently reported a PS OCT based method to quantify the double pass phase retardation induced strictly by the Henle fiber layer. On three patients, the study showed elevated double-pass retardation of 20°-to-23° occurring at an average retinal eccentricity of ca. 1.8° (range 1.5° to 2.25°). The method was also able to determine the fast axis of retardation. These results were consistent with previous knowledge of the radial pattern of Henle fibers.

Birefringence changes polarization in a predictable manner, which can be described by either the Mueller [154] or Jones [155] matrix of a linear retarder. A good review of PS OCT is given in [148].

### Retinal identification using retinal scanning

The amount of birefringence can drop locally if a blood vessel is encountered during retinal scanning.This enables retinal identification for biometric purposes [109,156]. A circular scan of of 20° around the optic disc catches all major blood vessels entering the fundus through the disc. The blood vessels often displace the nerve fibers in the RNFL, and since they are not birefringent, a steep drop (‘blip’) in the signal proportional to the size of the blood vessel is observed. Similar drops are seen on GDx images, where the blood vessels are represented as dark lines on the bright RNFL background. The birefringence-based retinal identification has certain advantages over the more traditional light absorption method [157,158], such as using near-infrared light (NIR) which does not cause discomfort or pupil constriction to the test person the way visible (green) light does with absorption methods.

## Discussion and conclusions

As Table 1 shows, different imaging techniques allow obtaining of different types of diagnostic information from the retina. Compared to fundus imaging, scanning technologies are generally applied to smaller portions of the retina (smaller FOV), but allow higher resolution and depth-penetration. TD OCT scanning technologies were generally slower than fundus imaging. But with the latest developments in FD OCT and SS OCT, image acquisition times have been shortened, allowing applications also on pediatric patients. At the same time, with moving towards larger wavelengths (above 1 μm), depth penetration has increased significantly. In addition, increasing the bandwidth of the source and the speed of the axial scan has led to a significant improvement in depth (axial) resolution. It should be mentioned, that the ability of imaging cellular-level information, such as cone photoreceptors, depends on additional factors, such as image stabilization features, numerical aperture (influencing the lateral resolution), scanning speed etc.

Retinal scanning technologies have revolutionized ophthalmic diagnostics in the last few decades, improving decisively the ability to detect and follow progression of eye diseases like glaucoma, AMD, amblyopia etc. Current research effort is directed towards improving speed and resolution, in order to enable data acquisition and 3D reconstruction of retinal substructures. Newer technologies are expected to deliver more affordable instrumentation and thus reduce health care costs. Polarization sensitive technologies are expected to enhance contrast and specificity in identifying structures in OCT images by detecting induced changes in the polarization state of light reflected from the retina or cornea.

Retinal scanning methods are being used not only for obtaining diagnostic information from the retina through imaging. A growing trend is to use such methods for screening – for example screening for amblyopia [102-105] and screening for retinopathy of prematurity (ROP) through assessment of vascular characteristics [159]. Non-retinal applications of ophthalmic imaging technologies include non-contact biometry, identification and monitoring of intraocular masses and tumors, and elucidation of abnormalities in the cornea, iris, and crystalline lens, all at micrometer resolution [120]. The Duke group has recently developed a handheld OCT device for use in infants [160-162]. The same group reported intraoperative use of an OCT device for imaging during macular surgery [163].

A number of diagnostic instruments have been developed combining different modalities. Some of them are commercially available – i.e. the CIRRUS photo system of Carl Zeiss Meditec, combining their CIRRUS HD-OCT with a fundus camera, and the SPECTRALIS instrument from Heidelberg Engineering, combining SD OCT with cSLO.

Retinal birefringence scanning can be used in a variety of medical and non-medical applications, such as detection of central fixation, eye alignment, biometric devices, eye tracking etc.

## Abbreviations

ADHD: Attentionm deficit and hyperactivity disorder; AMD: Age-related macular degeneration; AO: Adaptive optics; AOSLO: Adaptive optics scanning laser ophthalmoscope; CCD: Charged-coupled device (here used for a camera); cSLO: Confocal scanning ophthalmoscope; CTIS: Computed tomographic imaging spectrometer; DSP: Digital signal processor; ECC: Enhanced corneal compensation; FCC: Fixed corneal compensator; FD OCT: Fourier domain optical coherence tomography; FPA: Focal plane array; FOV: Field of view; FWHM: Full width at half maximum (wavelength range); fs: Scanning frequency (RBS); GDx: SLP-based nerve fiber analyzer (developed by Laser Diagnostic Technologies and marketed later by Carl Zeiss Meditec); GDxVCC: The GDx with a variable corneal compensator; Hb: Deoxygenated hemoglobin; HbO2: Oxygenated hemoglobin; HRT: Heidelberg Retinal Tomograph; HSI: Hyperspectral imaging; IMS: Image mapping spectroscopy; NA: Numerical aperture; NEB: The noise equivalent bandwidth of the electronic filter used to demodulate the OCT signal; NIR: Near-infrared light; NPBS: Non-polarizing beam splitter; OCT: Optical Coherence Tomography; PBS: Polarizing beam splitter; PS: Polarization-sensitive; PSD: Polarization-state detector; PSF: Point-spread function; PS OCT: Polarization-sensitive Optical Coherence Tomography; QWP: Quarter-wave plate (having a retardance of λ/4); RBC: Red blood cells; RBS: Retinal birefringence scanning; RNFL: Retinal nerve fiber layer; ROP: Retinopathy of prematurity; S0: S_1_, S_2_, S_3_, Elements of the Stokes vector, describing the polarization state of light; SD OCT: Spectral domain optical coherence tomography (same as FD OCT); SLD: Superluminescent light emitting diode; SLO: Scanning laser ophthalmoscope; SLP: Scanning laser polarimeter / scanning laser polarimetry; SS OCT: Swept Source Optical Coherence Tomography; TD OCT: Time domain optical coherence tomography; TSLO: Tracking scanning laser ophthalmoscope; TSNIT: Temporal-Superior-Nasal-Inferior-Temporal maps, displaying the thickness values of the retina around the optic nerve; UHR OCT: Ultrahigh-resolution OCT; VCC: Variable corneal compensator.

## Competing interests

The author declares that he has no competing interests.

## Authors’ contributions

BG did the literature search, conceived the review, and wrote the manuscript.

## Authors’ information

The author’s original background is in biomedical engineering, electrical and computer engineering, and signal processing. He has been with the Wilmer Eye Institute at Johns Hopkins since August 2000, developing instrumentation for retinal birefringence scanning for medical applications. In the last 14 years he acquired knowledge and developed skills in ophthalmology, optics, optoelectronics, modeling of polarization-sensitive systems, and prototype development in the field of ophthalmic optics. He is collaborating with researchers at other universities, working in the same area.
